# Host and geographic barriers shape the competition, coexistence, and extinction patterns of influenza A (H1N1) viruses

**DOI:** 10.1002/ece3.8732

**Published:** 2022-03-21

**Authors:** Chaoyuan Cheng, Marcel Holyoak, Lei Xu, Jing Li, Wenjun Liu, Nils Chr. Stenseth, Zhibin Zhang

**Affiliations:** ^1^ State Key Laboratory of Integrated Management on Pest Insects and Rodents in Agriculture Institute of Zoology Chinese Academy of Sciences Beijing China; ^2^ CAS Center for Excellence in Biotic Interactions University of Chinese Academy of Sciences Beijing China; ^3^ Department of Environmental Science and Policy University of California Davis California USA; ^4^ 12442 Ministry of Education Key Laboratory for Earth System Modeling Department of Earth System Science Tsinghua University Beijing China; ^5^ CAS Key Laboratory of Pathogenic Microbiology and Immunology Institute of Microbiology Chinese Academy of Sciences Beijing China; ^6^ Savaid Medical School University of Chinese Academy of Sciences Beijing China; ^7^ 6305 Centre for Ecological and Evolutionary Synthesis (CEES) Department of Biosciences University of Oslo Oslo Norway

**Keywords:** coexistence, competition, geographic barrier, host barrier, influenza A virus, interspecific transmission

## Abstract

The influenza virus mutates and spreads rapidly, making it suitable for studying evolutionary and ecological processes. The ecological factors and processes by which different lineages of influenza compete or coexist within hosts through time and across geographical space are poorly known. We hypothesized that competition would be stronger for influenza viruses infecting the same host compared to different hosts (the Host Barrier Hypothesis), and for those with a higher cross‐region transmission intensity (the Geographic Barrier Hypothesis). Using available sequences of the influenza A (H1N1) virus in GenBank, we identified six lineages, twelve clades, and several replacement events. We found that human‐hosted lineages had a higher cross‐region transmission intensity than swine‐hosted lineages. Co‐occurrence probabilities of lineages infecting the same host were lower than those infecting different hosts, and human‐hosted lineages had lower co‐occurrence probabilities and genetic diversity than swine‐hosted lineages. These results show that H1N1 lineages infecting the same host or with high cross‐region transmission rates experienced stronger competition and extinction pressures than those infecting different hosts or with low cross‐region transmission. Our study highlights how host and geographic barriers shape the competition, extinction, and coexistence patterns of H1N1 lineages and clades.

## Graphical abstract

Using available sequences of influenza A (H1N1) virus in public database we identified six lineages of H1N1 and twelve clades with several replacement events for human‐hosted lineages. We found the estimated co‐occurrence probability of lineages sharing the same host is much lower than those sharing different hosts, and human‐hosted lineages had lower co‐occurrence probability and genetic diversity than swine‐hosted lineages. Our results indicate that H1N1 lineages sharing the same host or having a higher cross‐region transmission intensity experienced a higher competition and extinction pressure.

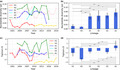

## 1 INTRODUCTION

Influenza A virus (IAV) is one of the most common pathogens worldwide and has caused massive damage to poultry production and human health for centuries (Morens & Taubenberger, 2011; Saunders‐Hastings & Krewski, 2016). The 1918 influenza pandemic (i.e., Spanish flu) and 2009 swine flu pandemic (pdm09) were caused by the H1N1 virus, accounting for more than 40 million deaths (Krammer et al., 2018; Palese et al., 2006). The IAV genome has eight single‐stranded RNA fragments, which cause IAV to mutate rapidly (Neumann et al., 2009; Webster et al., 1992). H1N1 is an RNA virus that mutates quickly, with an overall mutation rate of about 1.8 × 10^−4^ s/n/r (substitutions per nucleotide per strand copied; Pauly et al., 2017). As a consequence of the high mutation rate, there are many subtypes of IAV (e.g., H1N1, H3N2), based on hemagglutinin (HA) and neuraminidase (NA). The H1N1 subtype IAVs have long been circulating in humans, swine, and birds.

H1N1 can be categorized according to evolutionary relationships, with a strain representing virus populations with the same genome sequence, and strains with a common ancestor representing clades and lineages representing more distant common ancestry among clades. The eight segments that make up the influenza virus genome can be recombined with segments of other strains, clades, lineages, or subtypes through a process called reassortment or genetic shifts that rapidly produce new subtypes (Krammer et al., 2018). In recent decades, most outbreaks of influenza pandemics have resulted from new subtypes produced by reassortment (Kawaoka et al., 1989; Smith et al., 2009). The H1N1 virus has the longest spread history of all existing subtypes and has caused pandemics many times (Krammer et al., 2018). Thus, it is a suitable organism for studying the evolution and extinction patterns of organisms.

The H1N1 viruses mainly spread during cold seasons (Cheng, Li, et al., 2021), and it has been recognized that the seasonal H1N1 viruses in humans are replaced quickly by new pandemic strains (Pica et al., 2012). The prevailing view is that influenza evolution is driven by the balance between host immune responses and virus mutations. Intensive selection imposed by the host immune system drives antigenic drift in the influenza virus and most strains die off in a short time, resulting in continuous replacement of old strains with new strains (Ferguson et al., 2003; Webster et al., 1992). The interaction of antigens within the host immune network and the spread of multiple strains could trigger cross‐immunization (Recker et al., 2007; Uekermann & Sneppen, 2016). Consequently, the emergence of novel pandemic strains would cause a cross immune response in hosts, which could cause the extinction of circulating seasonal influenza viruses (Pica et al., 2012). Hence, competition for “antigenic space” and for “breaking the existing herd immunity” of hosts by circulating seasonal strains of influenza virus should play a crucial role in shaping viral evolution and extinction patterns (Recker et al., 2007). However, the ecological processes and factors mediating competition, coexistence, and extinction of the influenza virus have been infrequently investigated.

Hosts may act as a barrier of interspecific transmission for influenza viruses. Host diversity represents the niche breadth of the virus, which is mainly constrained by the genetic differences among host species in antigen resources, cell receptors, or immune responses (Kuiken et al., 2006; Matrosovich et al., 2004; Nelli et al., 2010). Currently, circulating swine H1N1 virus is mainly divided into three lineages: classical swine lineage, human seasonal lineage, and Eurasian avian lineage (Anderson et al., 2016). However, some H1N1 lineages can be transmitted between hosts such as humans, swine, and birds (Krammer et al., 2018). The 2009 influenza A (H1N1) pandemic was caused by an emerging strain that shifted hosts from swine to humans after genome reassortment among three strains (Vijaykrishna et al., 2010).

Regions with different ecological factors are likely important drivers for the spread and evolution of H1N1 lineages. Previous studies have shown that the occurrence and spread of different subtypes or lineages will have regional differences (Anderson et al., 2016; Bedford et al., 2015; Krammer et al., 2018; Lycett et al., 2019; Su et al., 2015). Geographical barriers can reduce the competition among different H1N1 lineages; however, frequent human transportation can reduce the geographic barrier effect and increase the competition between lineages. Human mobility is more frequent and is often facilitated by long‐distance transport such as on planes or trains (Brownstein et al., 2006; Cheng, Li, et al., 2021). In contrast, the mobility of farm animals (pigs, chickens) is often restricted to short distances and local transportation modes (e.g., many countries require that poultry are slaughtered before they are transported among provinces). Previous studies have shown that human mobility contributes to the global dynamics of H3N2 influenza viruses (Lemey et al., 2014). Our previous study indicated that human‐hosted influenza viruses (H1N1, H3N2) showed much higher spreading velocity and longer distance transmission distance than avian‐hosted influenza viruses (H7N9, H5N1), which might be transmitted by poultry or wild birds (Cheng, Li, et al., 2021). The smaller cross‐region mobility of farm animals may contribute to the observed lower replacement rate of old influenza strains by novel ones in swine than humans.

Species coexistence could be largely explained by classic ecological niche theory (Armstrong & McGehee, 1980; Grinnell, 1917; Holt, 2009; Hutchinson, 1957; Johnson, 1910; Pearman et al., 2008).

**FIGURE 1:**
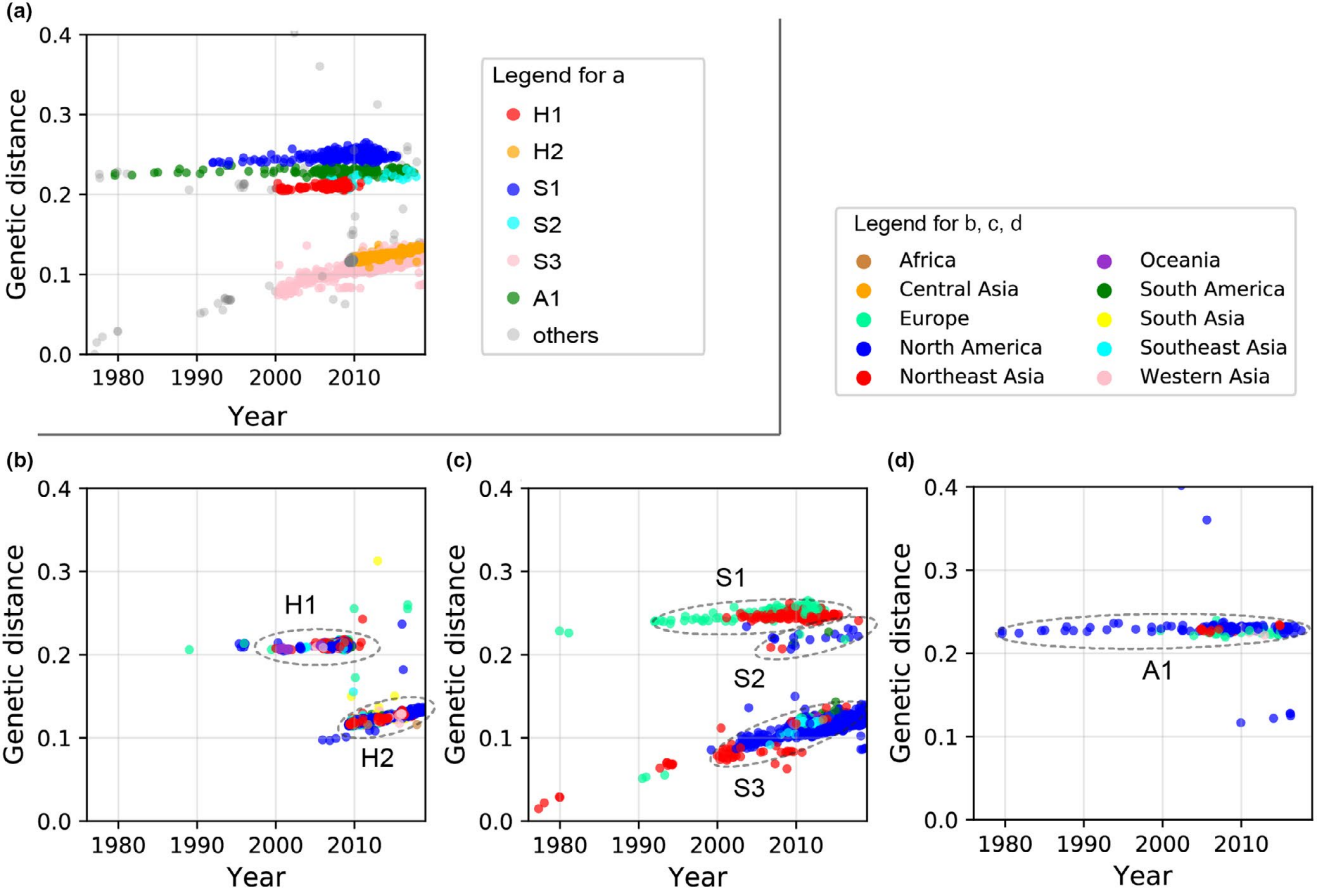
Genetic distance versus time of six H1N1 lineages and their geographical occurrence. (a) Six H1N1 lineages from all samples of different hosts. (b) Two lineages from human‐host samples in different regions. (c) Three lineages from swine‐host samples in different regions. (d) One lineage from avian‐host samples in different regions. Dots in (a‒d) represent samples (isolates), and their colors indicate sampling regions (b‒d) or hosts (a). Genetic distance represents the genetic distance from the oldest sample (A/swine/Hong Kong/61/1977) of our data. Elliptical dashed lines in (b‒d) indicate lineages

**FIGURE 2:**
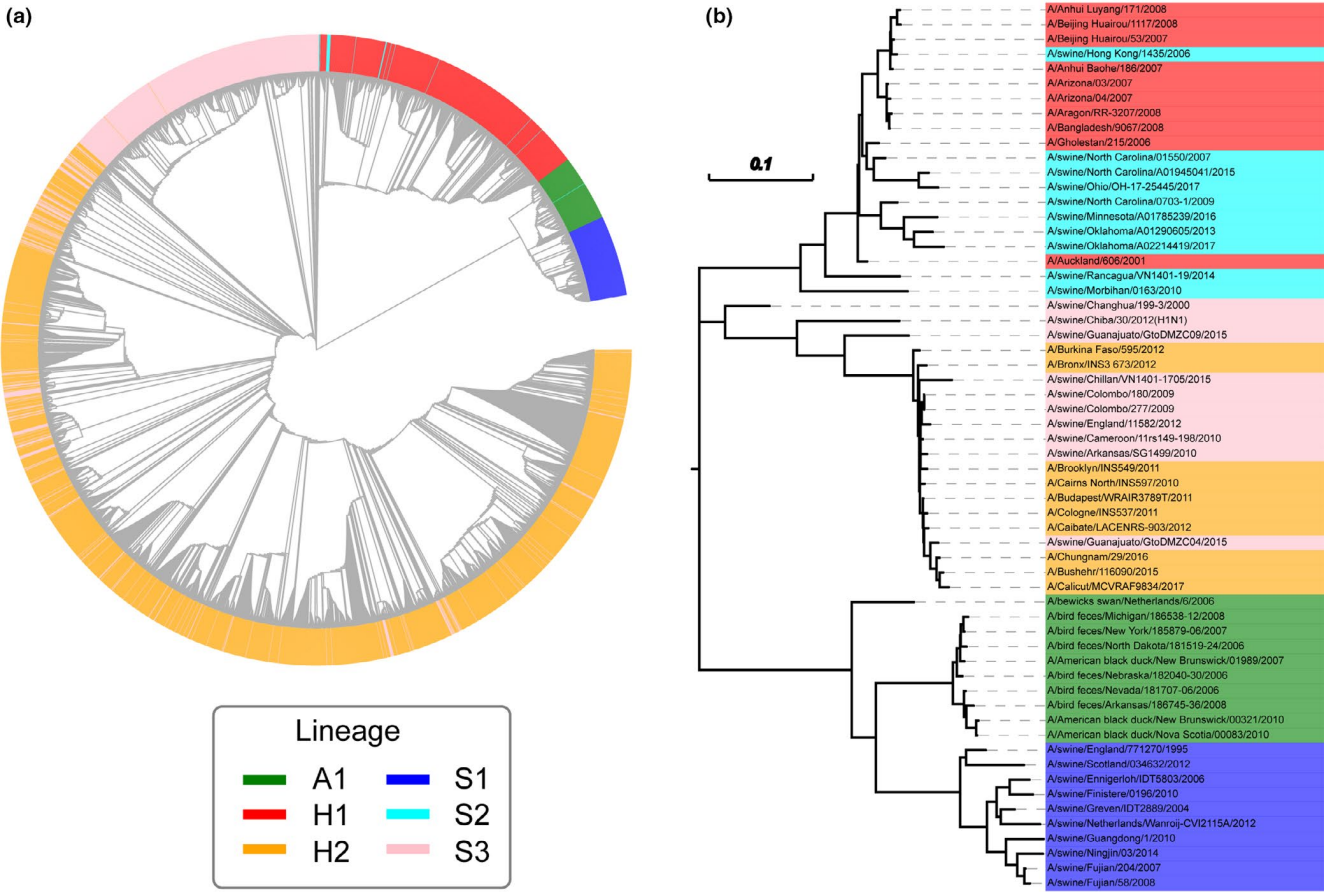
Phylogeny of HA genes using the combined dataset of six H1N1 lineages. (a) was generated from all identified samples, and (b) was generated from randomly selected samples (10 samples per lineage). Different colors indicate different lineages. H1, H2: human‐hosted lineages. S1, S2, S3: swine‐hosted lineages. A1: avian‐hosted lineage

We used available H1N1 HA gene sequences in GenBank (https://www.ncbi.nlm.nih.gov), to analyze the evolution and extinction patterns of H1N1 from different hosts (human, swine, and birds) and different regions, and estimated the co‐occurrence probability of different lineages. According to niche theory, species that share the same resources or have more overlap in space and time will compete more severely than species that use different resources or are separated. Specialized utilization of resources or geographic barriers could reduce species competition and increase the likelihood of species coexistence (Hardin, 1960; Pacala & Roughgarden, 1985). We use the term “competition” to refer to indirect competition through mechanisms including host immunity. Thus, we hypothesize the following: (1) H1N1 lineages infecting the same host will experience higher competition pressures than lineages with different hosts, which will result in higher lineage replacement rates and lower genetic diversity (i.e., the Host Barrier Hypothesis). (2) H1N1 lineages with a higher cross‐region transmission intensity will experience a higher competition pressure than those with a lower cross‐region transmission intensity; this will result in higher lineage replacement rates and lower genetic diversity with greater cross‐region transmission rates (i.e., the Geographic Barrier Hypothesis). In short, within any given host species, competition will prevent all lineages circulating at the same time and in the same region. In this study, lineages are defined by host and evolutionary unit (Figures 1 and 2). Lineages infecting the same host may belong to different evolutionary units (e.g., human lineage H1 and H2 in Figures 1 and 2), while lineages infecting multiple hosts may belong to the same evolutionary unit (e.g., the human lineage H1 and swine lineage S2 in Figures 1 and 2 belong to the same lineage). Evolutionary units were determined by phylogenetic methods (see below methods, Figure 2). Based on these hypotheses, we make the following predictions: (1) H1N1 lineages infecting the same host (e.g., in human lineages such as human lineage H1 and H2, or swine lineage S1, S2, and S3 in Figures 1 and 2) will have a smaller co‐occurrence probability than those infecting different hosts (e.g., between human lineage and swine lineage); (2) human‐hosted H1N1 lineages will have a lower co‐occurrence probability and genetic diversity than swine‐hosted lineages.

## 2 METHODS

### 2.1 Sequences

We downloaded the sequence data of H1N1 IAV from GenBank (https://www.ncbi.nlm.nih.gov/) using a custom Python script with the "Biopython" package (https://biopython.org/) and extracted the sequences encoding Hemagglutinin (HA). The dataset includes 32,759 records of H1N1 viruses, including information on sampling locations and dates for each report. We assigned the sampling location with the latitude and longitude based on the administrative center coordinates using the "GeoPy" package (Esmukov, 2020) in Python 3.6.0 (https://www.python.org/).

We excluded samples that lacked a specific day of sampling or strain codes, as well as those with sequence length <1600 bp in length (to ensure the sequences were complete HA sequences, making up 29.8% of the total sequences). To reduce the impacts of sampling effort bias on the research, we only used one sample from the same place in the same month (25.9% of total sequences were removed). Finally, 6097 samples of the H1N1 virus from 1279 locations were used. For subsequent analysis at the amino acid codon level, all sequences were aligned using an H1N1 sequence (A/swine/Hong Kong/61/1977) as a template sequence and to remove all gaps and redundant bases corresponding to the template sequence to be able to convert the sequence to codons (which enables the conversion of nucleotide sequences into amino acid sequences). Sequences were aligned using MEGA7 (Kumar et al., 2016) with default parameters.

### 2.2 Identification of H1N1 lineages

We classified the cleaned sequences into three host types: human, swine, and avian. We calculated the genetic distance (GD) between each cleaned sequence and the earliest sampled sequence (A/swine/Hong Kong/61/1977) separately. Then we plotted GD versus sampling date separately for each host type. Based on the molecular clock theory (King & Jukes, 1967; Kimura, 1968), the mutation rate of HA can be considered constant, and six lineages were readily identified using linear relationships between GD and sampling time (genetic distance and sampling time method, or GD‐time method; see Figure 1a). According to hosts they infect, influenza A (H1N1) viruses can be classified into three categories: human, swine, and avian influenza, and transmission of influenza viruses of the same category mostly occurs among members of the same host species (Matrosovich et al., 2004; Nelli et al., 2010; van Riel et al., 2007). Thus, we use the following criteria to identify the lineage of the samples: (1) samples with the same host; (2) samples which form a close and continuous linear cluster on the two‐dimensional space by genetic distance (GD) and sampling time. Samples from the lineage defined here should belong to a unique evolutionary unit which was further verified by using the phylogenetic tree (see below). The GD‐time method has the advantage of revealing the replacement of lineages or interspecific transmission events in time (Figure 1), while using the phylogenetic tree has the advantage of revealing the distinct evolutionary units (Figure 2).

### 2.3 Evolution of H1N1 clades

To identify the distinct evolutionary units of H1N1 and clades of each H1N1 lineage, we performed Bayesian phylogenetic analysis in BEAST 2.6.2 (Bouckaert et al., 2019) for each H1N1 lineage we identified above. Sampling dates were used as “tip dates” (i.e., occurrence time of the viruses), and sampling location was used as a discrete trait. All analyses were performed using a coalescent model with a strict molecular clock and constant population size. In each analysis, the length of the Markov chain was set to 1 × 10^8^, with sampling every 1000 steps. The significance of the BEAST analysis was assessed in Tracer (software included with BEAST), and the effective sample size (ESS) of major parameters was >200 after removing 10% of the chain as burn‐in. Maximum clade credibility (MCC) trees annotated with the discrete trait (region) were generated in TreeAnnotator (software included with BEAST).

### 2.4 Cross‐region transmission of H1N1 lineages

All samples of the six identified H1N1 lineages were divided into nine major regions according to sampling locations: North America (2965 samples), South America (204 samples), Europe (351 samples), Africa (46 samples), Oceania (84 samples), Western Asia (150 samples), Northeast Asia (568 samples), Southeast Asia (187 samples), and South Asia (226 samples). Due to the large size of Asia and the existence of many geographical barriers, we divided Asia into five regions consisting of the four listed above and Central Asia according to the *M49 standard* (https://unstats.un.org/unsd/methodology/m49/), but there were no samples from Central Asia. We performed a Bayesian discrete phylogenetic analysis in BEAST 1.10.4 (Drummond et al., 2012) to reconstruct the ancestral state of each node in the phylogenetic tree for the discrete trait (region) using resampled data. The trait substitution model was set to be asymmetric. We applied Bayesian stochastic search variable selection (BSSVS) to estimate the significance of pairwise transitions between regions for each analysis. We used Bayesian Factor (BF) as a measure of statistical significance (Lemey et al., 2009), computed in SpreaD3 (Bielejec et al., 2016). Inferences from the BSSVS analysis may be influenced by the different sample sizes in each region (De Maio et al., 2015). Thus, to reduce the bias, we used resampled data in the BSSVS analysis. In resampled data, 10 samples per year per region per lineage were retained (if there were less than 10 samples, all samples were retained).

For each H1N1 lineage, we built a network by treating regions as the nodes. Two nodes were regarded as connected if BF‐values between them were greater than 3, indicating strong support for the cross‐regional transmission (Lemey et al., 2009). We calculated the degree, degree centrality, closeness centrality, and betweenness centrality of each network with the NetworkX package (Hagberg et al., 2008) in python 3.6.0.

### 2.5 Selection pressure (d_N_/d_S_), nucleotide diversity, and Tajima's *D*

The selection pressure on a virus is closely related to its mutation rate (Frost et al., 2018). To compare the selection pressures on different H1N1 lineages, we calculated the ratio of nonsynonymous and synonymous (d_N_/d_S_) mutations in the data of each lineage with the SLAC (Kosakovsky Pond & Frost, 2005) method in HyPhy 2.5.14 (Kosakovsky Pond et al., 2020) and Datamonkey (Weaver et al., 2018).

To measure the extinction pressure of an H1N1 lineage indirectly, we calculated the nucleotide diversity (pi) and Tajima's *D* of each lineage for each year using custom python scripts in the "DendroPy" package (Sukumaran & Holder, 2010). Larger pi and Tajima's *D* values mean that extinction events are rarer. Since influenza occurrence has apparent seasonality, we used the period from July 1 to June 30 of the following year to represent a year (e.g., data from July 1, 2001 to June 30, 2002 represent the year 2002) to perform the following estimated co‐occurrence probability calculations.

### 2.6 Estimated co‐occurrence probability between lineages

To quantify the estimated co‐occurrence probability of different lineages from different hosts in the same year and region, we defined species co‐occurrence probability in a time‐space dimension by following our previous studies (Yan et al., 2016):

(1) $$C_{i,j}=1‐0.5\sum_{k=1}^{n}\left|P_{\mathrm{ik}}‐P_{\mathrm{jk}}\right|.$$

Here, $C_{i,j}$ is the co‐occurrence probability between lineage *i* and lineage *j* in a specific year and region. We defined samples of lineages *i* and *j* as co‐occurring when they were collected from the same year and same region. *k* represents a specific year and a specific region (year‐region), *n* represents the total number of all possible time and region combinations for lineage *i* and lineage *j*. For example, if the samples cover ten years and nine regions; thus, *n* = 10 × 9 = 90. $P_{\mathrm{ik}}$ is the proportion of the number of samples of lineages *i* in the year‐region *k* to the number of all samples in the year‐region *k*. The smaller $C_{i,j}$ indicates that the two lineages had little probability of co‐existence.

Since there is only one avian‐hosted lineage, we only calculated co‐occurrence probability for swine and human lineages. For human lineages (*H*1, *H*2), we calculated $C_{H1,H2}$, which represents the co‐occurrence probability between the human‐hosted lineages. For swine lineages, because the sample size of S2 is small, we only calculated $C_{S1,S3}$, which represents the co‐occurrence probability between the swine lineages. We also calculated $C_{S,H}$, $C_{S,A}$ and $C_{H,A}$, which represents the co‐occurrence probability of H1N1 lineages from different hosts.

To test whether two lineages tended to be exclusive or inclusive with each other in term of co‐occurrence probability in the year and location, we designed a computer simulation algorithm based on stochastic process (Tijms & Tijms, 1994) to calculate the distribution of $C_{i,j}$ under the null hypothesis without exclusion or inclusion in co‐occurrence (i.e., random co‐occurrence between them). The simulation algorithm is defined as follows:

(2) $$Sim\left(i,j\right)=rand\left(t_{\left(i,j\right)},r_{\left(i,j\right)}\right).$$

Here, Sim(i,j) is the co‐occurrence probability of lineage *i* and *j* from randomly simulated lineages. $t_{\left(i,j\right)}$ and $r_{\left(i,j\right)}$ is the total occurrence time and location ranges of lineage *i* and *j*. $rand\left(t_{\left(i,j\right)},r_{\left(i,j\right)}\right)$ represents randomly generating an attribute in the time period $t_{\left(i,j\right)}$ and location range $r_{\left(i,j\right)}$. For each lineage, we generated the same number of simulated attributes as the sample size of the lineage and then calculated the simulated co‐occurrence probability according to Equation 1. We calculated 1,000,000 simulated co‐occurrence probabilities between lineages. Calculations of co‐occurrence probability and simulation algorithms were implemented using custom python scripts (see Data Availability Statement section).

## 3 RESULTS

### 3.1 Identification of major H1N1 lineages

The evolutionary patterns resulting from employing the genetic distance and sampling time (GD‐time) approach are shown in Figure 1 and those from the phylogenetic tree method are shown in Figure 2. We identified six main H1N1 lineages: two human (H1, H2; Figure 1b), three swine (S1, S2, S3; Figure 1c), and one avian lineage (A1; Figure 1d). The lineages we identified are generally consistent with previously recognized lineages (Anderson et al., 2016). Based on the HA gene, the H1 and S2 lineages belong to the Human Seasonal Lineage. The H2 and S3 lineages belong to the same evolutionary unit as the Classic Swine Lineage and the S1 lineage is similar to the Eurasian Avian Lineage (Table 1, Figure S1). The S3 and H2 lineages are continuous and overlapped in the genetic distance (GD)‐time dimension. S3 appeared earlier than H2 (Figure 1a), which is consistent with interspecific transmission from the swine (i.e., S3 lineage) to human (i.e., H2 lineage) lineage, causing the H1N1 pandemic in northern America in 2009 (Krammer et al., 2018; Neumann et al., 2009). Similarly, H1 and S2 overlapped in the GD‐time dimension but H1 appeared earlier (Figure 1a), which indicates another interspecific transmission of H1N1 from humans (i.e., H1 lineage) to swine (i.e., S2 lineage). This transmission has not been reported before. Notably, the H1 lineage disappeared immediately after the appearance of the H2 lineage, but S1, S2, and S3 have considerable overlap in time and space (Figure 1). Lineages from different hosts (human, birds, swine) also had considerable overlap in both time and space dimensions. These patterns are consistent with the Host Barrier Hypothesis.

**TABLE 1:** Relationship between H1N1 lineages we defined and the traditional lineages defined in other studies (also see Figure S1). “Appearance year” represents the circulating time of each lineage in this study

	A1	H1	H2	S1	S2	S3
Traditional lineage	None	Human seasonal lineage	Classic swine lineage	Eurasian avian lineage	Human seasonal lineage	Classic swine lineage
Appearance year	1979–2017	2000–2010	2010–2018	1992–2015	2006–2017	2000–2018

Phylogenetic analysis also supported the above observations about lineage identification and host associations. The A1 and S1 lineages are relatively independent, with little genetic similarity to other lineages (Figure 2a,b). The H2 and S3 lineages have many similar strains, and the S2 lineage has many strains similar to the H1 lineage (Figure 2a,b). These results support the observation that interspecific transmission events of H1N1 lineages occurred between swine and humans.

The stop codons of all samples in A1 are TAG. However, the stop codons of other lineages are mainly TAA (Table 2), indicating that A1 is older than the common ancestor of other lineages. Compared with the other lineages, H1 and S2 have a deletion of three bases (AAA) in the same nucleotide position (439‒441), resulting in a lack of one amino acid (Lysine) (Table 2), further supporting the observation that S2 is derived from H1.

**TABLE 2:** Stop codon types and codon deletion in different lineages. “Yes” in the table indicates a lineage that lacked three bases (AAA) at positions 439, 440, and 441 of the corresponding template nucleotide sequence

	A1	H1	H2	S1	S2	S3
Stop codon	TAG	TAA	TAA	TAA	TAA	TAA
Deletion	No	Yes	No	No	Yes	No

### 3.2 Time‐scaled evolution of the H1N1 clades

**FIGURE 3:**
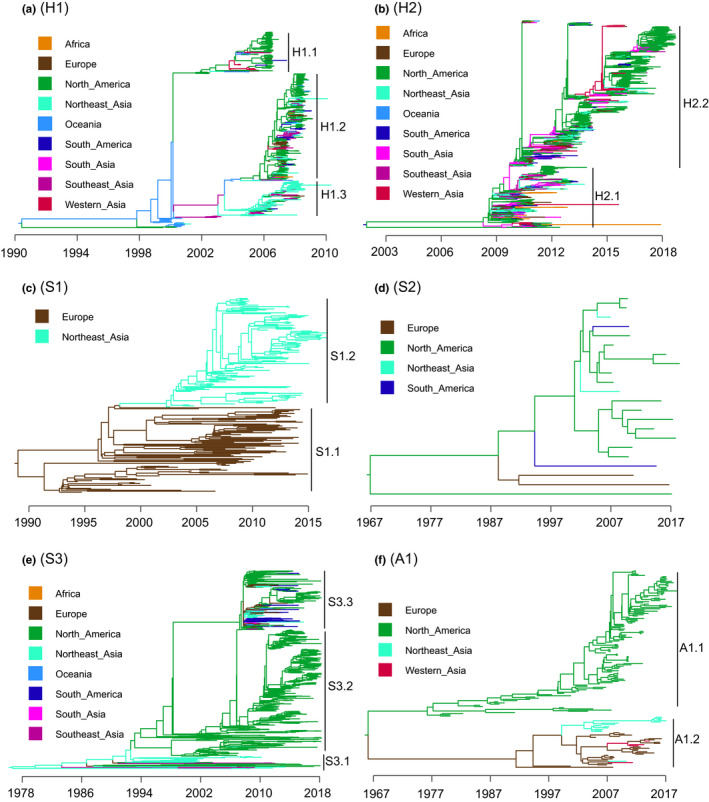
Bayesian time‐scaled trees of the six H1N1 lineages (HA genes). Different colors indicate different regions. (a, b) human‐hosted lineages (H1, H2). (c, d, e) swine‐hosted lineages (S1, S2, S3). (f) avian‐hosted lineage (A1)

Analysis using Bayesian time‐scaled trees revealed the finer‐scale evolutionary patterns of H1N1 lineages. The H1 lineage can be further divided into three main clades (H1.1, H1.2, H1.3). H1.1 disappeared around 2007, while H1.2 and H1.3 occurred around 2007 and then disappeared in 2009. For H1 lineage, the main prevalent clades in North America are H1.1 and H1.2, while the prevalent clade in Northeast Asia is H1.3 (Figure 3a). It is notable that the observed patterns are caveated by the fact that lack of data from some parts of the world such as from South America, Oceania, and Africa. The distribution regions of H1.1 and H1.2 overlap a lot and H1.1 is replaced geographically by H1.2 while H1.2 and H1.3 coexisted in different regions until they were replaced by the H2 lineage (Figure 1). The H2 lineage appeared around 2009 (Figure 1a) almost simultaneously in many regions of the world (Figure 3b), and is related to pdm09, which caused the 2009 H1N1 pandemic. This lineage is further classified as two main clades (H2.1, H2.2); H2.1 clade spread globally after the pdm09 outbreak, and H2.2 originated from a strain in H2.1, which replaced the other strains in H1.1 and is still circulating widely until now (Figure 3b).

The S1 lineage first appeared in Europe (S1.1) and later spread to Northeast Asia (S1.2) (Figures 1c and 3c). These two clades of S1 lineage stayed in two different regions and disappeared around 2016 (Figure 3c). The S2 lineage samples are relatively small (*n* = 22), first appeared and mainly occurred in North America (Figure 3d). The S3 lineage first appeared in Northeast Asia (S3.1), then spread in North America (S3.2) and globally (it produced S3.3) after 2009 (Figure 3e). The A1 lineage is divided into 2 clades (A1.1, A1.2), which separated from each other a long time ago. A1.1 spread only in North America, while A1.2 first occurred and mainly spread in Europe and then spread to Northeast Asia (Figure 3f).

Notably, the human‐hosted lineages (H1, H2) showed a clear pattern of clade replacement, indicating more frequent extinction of older clades. For example, H1.1 was replaced by H1.2, and H2.2 replaced H2.1. Such replacement was rare in the swine‐ and avian‐hosted lineages. H1 and H2 lineages (also A1.1) showed an apparent asymmetric evolution with one or several dominant clades (i.e. tree‐like evolution). In contrast, the other lineages (S1, S2, S3, and A1) showed more symmetric evolution with more old clades (i.e., bush‐like evolution). H1 showed prominent fast or explosive evolution (i.e., H1.1, H1.2) within a short period.

### 3.3 Cross‐region transmissions

**FIGURE 4:**
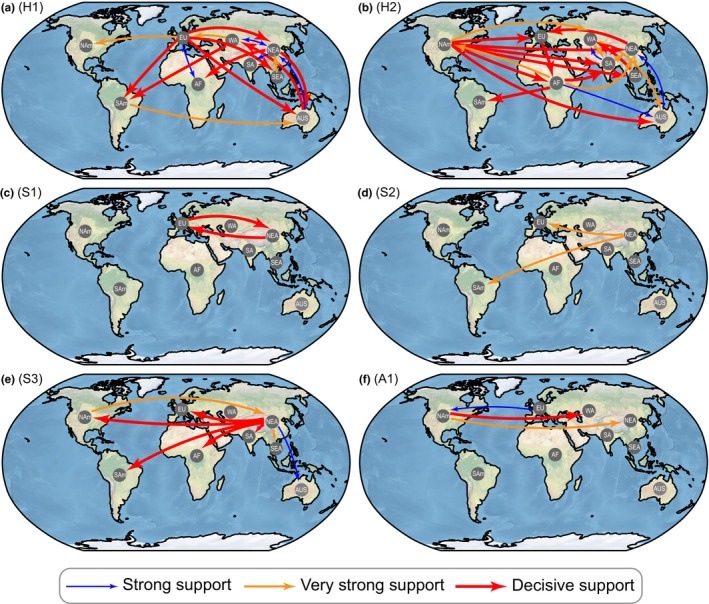
Cross‐region transmission intensity of H1 (a), H2 (b), S1 (c), S2 (d), S3 (e), and A1 (f) lineages based on resampled data. Arrows indicate significant cross‐regional spread of the virus among the two connected regions. Arrow color and thickness indicate transmission intensity: blue for strong support (Bayesian Factor (BF) > 10), orange for very strong support (BF > 30), and red for decisive support (BF > 100)

The human‐hosted H1 (Figure 4a) and H2 (Figure 4b) lineages have a higher cross‐region transmission intensity (as measured by the number and strength of transmission routes) around the world. Swine‐ and avian‐hosted lineages have a small cross‐region transmission intensity (except for S3). The swine‐hosted S1 lineage only transmitted between Europe and Northeast Asia, and the S2 lineage only transmitted from Northeast Asia to Europe and South America (Figure 4c,d). The S3 lineage has many cross‐region transmission routes around the world (Figure 4e). The avian‐host A1 lineage mainly was transmitted from North America to West Asia and Europe, and from Northeast Asia to North America (Figure 4f). These results indicate that the cross‐region transmission intensity order is that: H1, H2 > S3 > A1 > S1 > S2. Europe and Northeast Asia in the H1 lineage, Africa and North America in the H2 lineage, and Northeast Asia in the S3 lineage, had a higher degree centrality (>1). This indicates that these regions had a greater contribution to the spread of viruses in the corresponding lineages (Figure 4a,b,e; Table S1). Although the sample size of Africa in H2 is relatively small, the resampling results suggest that Africa may play a role in the cross‐regional spread of H2.

### 3.4 Estimated co‐occurrence probability among lineages

**FIGURE 5:**
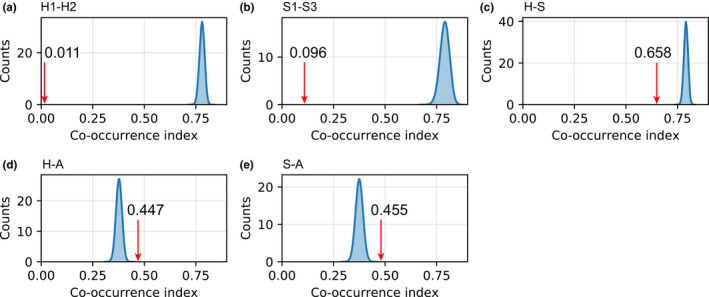
The observed (red arrow) and simulated (blue bell curve) co‐occurrence probabilities among the same host lineages (a, b) and among different host lineages (c, d, e). (a) H1 vs. H2; (b) S1 vs. S3; (c) Human vs. Swine; (d) Human vs. Avian; and (e) Swine vs. Avian

For both the human‐ and swine‐hosted lineages, the observed co‐occurrence probability was far less than simulated random co‐occurrence probabilities (Figure 5a–c). This indicates that competitive exclusion reduced the co‐occurrence among the lineages. However, the observed co‐occurrence probability between avian‐ and human‐ or swine‐hosted lineages was higher than randomly simulated co‐occurrences (Figure 5d,e). The estimated co‐occurrence probabilities (0.011‒0.096) within the same host (i.e., for either human‐ or swine‐host, Figure 5a,b) were much lower than those (0.45‒0.66) for different hosts (Figure 5c–e).

### 3.5 Selection pressure and genetic diversity

Although the HA genes of all lineages were subject to adverse selection, the A1 lineage suffered a stronger adverse selection (d_N_/d_S_ = 0.088; Table 3) compared to other lineages (d_N_/d_S_ > 0.15). The nucleotide diversity (pi) of the animal‐hosted lineages (A1, S1, S2, and S3) was much higher than that of the human‐hosted lineages (H1, H2; Table 3). H1, H2, and S3 lineages were under similar selection pressure, but the genetic diversity of H1 and H2 was significantly smaller than that of S3 (Table 3).

**TABLE 3:** The selection pressure (d_N_/d_S_) and nucleotide diversity (pi) of HA genes in each lineage

Lineage	Global d_N_/d_S_	Positive sites	Negative sites	Pi	Total sites	Number of sequences
H1	0.272	3	189	0.0248	566	695
H2	0.256	19	363	0.0156	566	2742
S1	0.216	1	224	0.0673	566	203
S2	0.16	1	160	0.1015	566	22
S3	0.235	19	429	0.0687	566	953
A1	0.088	1	325	0.0836	566	166

**FIGURE 6:**
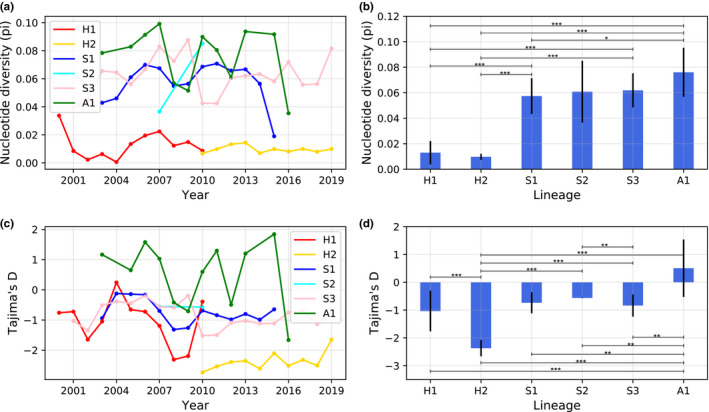
Extinction pressure as measured by nucleotide diversity (a, b) and Tajima's *D* (c, d) for each lineage. The lines in (a) and (c) represent the annual value and trend of each lineage, and the bars in (b) and (d) represent the mean value (error bars indicate the standard deviation of each lineage) over the years. The asterisk symbols in (b) and (d) represent the significance level of *t*‐test between lineages: ***p < .001. **.001 < *p* < .01, and *.01 < *p* < .05

Both pi (Figure 6a,b) and Tajima's *D* (Figure 6c,d) of human‐hosted H1N1 lineages are much smaller (indicating more frequent extinction) than those of swine‐ or avian‐hosted lineages (for most cases, *p* < .05, .01 or .001). The avian lineage A1 had the highest diversity, and its Tajima's *D* was positive (Figure 6), indicating few extinct events, and low selection pressure.

## 4 DISCUSSION

Although previous studies have suggested that competition between lineages is an important driver in shaping the ecology and evolution of the influenza viruses (Ferguson et al., 2003; Pica et al., 2012; Recker et al., 2007; Webster et al., 1992), the ecological factors and processes mediating competition, coexistence and extinction of influenza lineages are poorly known. In this study, we found that within any given host species, competition prevented all lineages circulating at the same time in a given region. H1N1 lineages infecting the same host species had a lower co‐occurrence probability than those infecting different host species. Furthermore, human‐hosted H1N1 lineages with a high cross‐region transmission intensity showed a lower co‐occurrence probability than swine‐hosted lineages with lower cross‐region transmission intensity, which supports our hypotheses and predictions.

### 4.1 Coexistence and extinction of H1N1 lineages under competition

The estimated co‐occurrence probability is widely used to represent interspecific competition strength (Yan et al., 2016). Smaller co‐occurrence probability often indicates more intense competition or frequent extinctions. Frequent extinction events in local populations will reduce genetic diversity (McCauley, 1991). The high replacement rate of influenza strains is attributed to the low genetic diversity of virus strains in humans (Ferguson et al., 2003). These results suggest that genetic diversity could be used as a proxy for the extinction rate.

Rapid replacement of old influenza lineages with novel ones in humans has been observed in previous studies (Anderson et al., 2016; Bedford et al., 2015; Krammer et al., 2018; Lycett et al., 2019; Su et al., 2015). For example, the normal circulating influenza virus strains disappeared after novel viruses emerged in 1957 and 1968 (Palese & Shaw, 2006; Palese & Wang, 2011). After the emergence of the 2009 H1N1 pandemic, the previously circulating swine H1N1 was replaced by the pandemic H1N1 during 2010–2011 (Pica et al., 2012). We found the estimated co‐occurrence probability of human‐hosted lineages was lowest (0.011) (Figure 5), indicating that competition among human‐hosted lineages is likely very high. Indeed, we found one lineage replacement event in human‐hosted lineages (i.e., H2 replaced H1) by the GD‐time method. Similar patterns were observed for the replacement events at the clade level: H1.1 was replaced by H1.2 and H1.3, and H2.1 was replaced by H2.2 (Figure 3a), which is consistent with previous observations (Palese & Shaw, 2006; Palese & Wang, 2011). However, the co‐occurrence probability of swine‐hosted lineages (S1, S2, S3) was relatively higher (0.096) (Figure 5), and we did not see such replacement through time in swine‐hosted lineages, indicating lower competition in swine‐hosted H1N1 lineages than in human‐hosted H1N1 lineages. Notably, the competition defined here refers to indirect competition similar to the “apparent competition” in ecology, that is, competition between two species via a third species. The presence of one H1N1 lineage could enhance the immunity of the host population (i.e., cross‐immunity) and thus exerts negative selection pressure on other nondominant lineages (Pica et al., 2012). Besides, isolation measures such as travel restrictions and social distance can be effective in controlling the spread of viruses (Cheng, Wan, et al., 2021). Thus, human interventions such as vaccination and isolation may also have similar effects in mediating indirect competition between H1N1 lineages.

Differences in infection or transmission efficiency between old and new lineages, driven by immune adaptation, can determine the outcome of lineage replacement (Ferguson et al., 2003). When a lineage of influenza virus irrupts in a host, the host's immune system is activated and exercised, making the host more resistant to the virus (Krammer et al., 2018). The newly outbreaking lineage has the advantages of larger infection volume and higher infection efficiency, which in the face of increased host immune response would cause the old lineages to face increased competition and pressure of extinction. Thus, as the level of herd immunity increases, the emerging lineages will gain a competitive advantage and thus replace the older lineages (Ferguson et al., 2003; Pica et al., 2012; Recker et al., 2007; Webster et al., 1992).

Competition strength is largely determined by the niche similarity between species (Hardin, 1960; Holt, 2009; Pearman et al., 2008; Pianka, 1974). Influenza viruses compete for limited antigen resources of hosts (Pica et al., 2012; Recker et al., 2007). Therefore, lineages sharing the same host would compete with each other more strongly by infecting the same host, which explains why the estimated co‐occurrence probability (0.011‒0.096) of human‐ or swine‐hosted H1N1 lineages was very low, as compared to those (0.447‒0.658) among different hosts (i.e., between human‐ and swine‐hosted lineages). Due to the high replacement rate (estimated by nucleotide diversity and Tajima's *D*) of strains in humans (Figure 6), driven by the cross‐region transmission, niche separation for H1N1 lineages in humans mainly occurred in time, whereas in swine, it occurred across space.

### 4.2 Host barriers and interspecific transmissions

Previous studies indicate that different influenza virus strains have distinct hosts (e.g., humans, pigs, birds) (Kuiken et al., 2006), suggesting there are obvious barriers between different hosts limiting interspecific transmission. The receptor binding, HA acid stability (e.g., the pH inside the host cell will affect the activation of HA), and polymerase activity are related to the interspecies transmission of IAVs (Herfst et al., 2014; Russier et al., 2017). Human‐adapted IAVs tend to bind to α‐2,6 receptors, while avian‐adapted IAVs tend to bind α‐2,3 receptors, and both receptors are abundant in pigs (Matrosovich et al., 2004; Nelli et al., 2010; van Riel et al., 2007). When the influenza virus enters the host cell, it needs to activate the HA protein at a specific pH to cause membrane fusion and release the viral genome into the cytoplasm of infected cells (Bullough et al., 1994). Avian influenza needs to activate HA protein at a higher pH, whereas human influenza needs to activate HA protein at a lower pH, and pigs support a broad range of HA activation pH (Russier et al., 2016, 2017). The host barrier, similar to food resource partition, could play a role of niche separation, thus reducing the competition strength and facilitating the coexistence of H1N1 lineages from different hosts. This explains the co‐occurrence probability between human‐ and swine‐hosted H1N1 lineages (0.447–0.658) higher than that among human‐hosted lineages (0.011) or swine‐hosted lineages (0.096).

However, interspecific transmissions are also seen due to mutation or re‐assortment of the influenza virus (Karakus et al., 2019; Kawaoka et al., 1989; Krammer et al., 2018; Landolt & Olsen, 2007). For example, the 2009 H1N1 pandemic was caused by an emerging strain from swine to humans due to the genome reassortment of three different strains (Vijaykrishna et al., 2010), which is supported by our observation on the interspecific transmission event from swine‐hosted lineage S3 to human‐hosted lineage H2 in 2009 (Figure 1). We also found another interspecific transmission event from human‐hosted H1 lineage to swine‐hosted S2 lineage (Figure 1). Notably, the two interspecific transmission events were continuous and smooth in genetic distance and time dimensions, suggesting the interspecific transmission rate was produced at a normal mutation rate.

### 4.3 Effects of geographic barriers and cross‐region transmissions

Geographic barriers are known to play a crucial role in the origin and evolution of a new species or subspecies by reducing gene flow (Howard, 2003; Zhao et al., 2021; Zhu et al., 2021). However, with the accelerated international movement of people and goods, species can spread easily across continents, which leads to serious biological invasion problems, such as the extinction of native species (Blackburn & Ewen, 2017; Suarez et al., 2001) as well as global transmissions of influenza virus (Cheng, Li, et al., 2021; Lemey et al., 2014). Previous studies have shown that geographic isolation prolonged the cocirculation of geographically segregated H1N1 lineages (Bedford et al., 2015). It is still unclear how cross‐region transmission of influenza viruses affects their competition and extinction patterns. Humans are much more capable of mobility than terrestrial animals, and can easily move across regions or continents through modern transportation (Brownstein et al., 2006). This may explain why human‐hosted H1N1 lineages (i.e., H1, H2) had a higher cross‐region transmission intensity than the swine‐hosted lineages observed in this study (e.g., S1, S2) (Figure 4). The high cross‐region transmission intensity of S3 is likely caused by its interspecific transmission with H2. High transmission intensity among geographic regions would impose a high competition pressure among lineages within the same host species, by reducing the geographic barrier effect and introducing novel variants. This likely explains why human‐hosted H1N1 lineages suffered a much higher competition or extinction pressure and much lower coexistence, as compared to swine‐hosted lineages. Previous studies have shown that the global circulation of H3N2 viruses is maintained by an East and Southeast Asian network that includes India, and most lineages of H1N1 viruses eventually coalesced with viruses from East and Southeast Asia and India (Bedford et al., 2015). According to our results, different lineages have different geographical transmission characteristics. Among the three global circulating lineages (i.e., H1, H2, S3), each of the H1 and H2 lineages had two significant transmission centers (i.e., H1: Europe and Northeast Asia; H2: Africa and North America), and Northeast Asia had a significantly higher contribution than other regions in global spread of S3 (Figure 4, Table S1). Notably, our analysis was based on lineages as defined based on hosts and evolutionary units (e.g., H1, H2, etc.) and not subtypes (e.g., H1N1, H3N2, etc.). Therefore, such competition patterns may differ between our lineages and those subtypes.

### 4.4 Genetic diversity and extinction rate

Genetic diversity is mainly determined by the effective population size and gene flow (Wang, 2002; Xu et al., 2013). It is recognized that gene flow could increase the genetic diversity of a population (Xu et al., 2013), but reduce heterogeneity among populations (Slatkin, 1987). In our study, we found human‐hosted H1N1 lineages had a higher cross‐region transmission intensity, but a lower genetic diversity, likely due to the extinction of clades under higher competition pressure of human‐hosted lineages. Lineages with high rates of clade extinction should have low genetic diversity. In our study, the nucleotide diversity and Tajima's *D* of swine‐ or avian‐hosted H1N1 lineages was significantly higher than that of human‐hosted lineages, indicating that human‐hosted H1N1 lineages suffered more extinction pressure than the swine‐ or avian‐hosted lineages. According to our study, the cross‐region transmission intensity of H1N1 lineages is very similar to the biological invasion process, which can cause massive extinction of local native species (Clavero & Garciaberthou, 2005). Our observations are consistent with the previous results that human H1N1 lineages have very low genetic diversity, high extinction rates, and that new strains regularly replaced old strains (Ferguson et al., 2003; Pica et al., 2012).

### 4.5 Evolutionary patterns

The lineages we identified are generally consistent with previously recognized lineages (Anderson et al., 2016). Genetically, the H1 and S2 lineages are similar to the Human Seasonal lineage, the H2 and S3 lineages are similar to the Classic Swine lineage and the S1 lineage is similar to the Eurasian avian lineage.

The H1N1 virus infects a large number of hosts and produces a large number of mutations every year. Although some major lineages have been classified (e.g., classical swine lineage, human seasonal lineage, Eurasian avian lineage), there is not a unified system to classify and name all H1N1 lineages and clades. Previous studies have shown that the Classical Swine lineage originated from the 1918 Spanish flu (Shope, 1931), while the Eurasian avian lineage (or Eurasian avian‐like) resulted from the spillover from avian flu in Europe with subsequent spread to Asia (Vincent et al., 2014), and the human seasonal lineage originated in Europe in the 1990s (Brown et al., 1995). In general, the relationship between H1N1 lineages and clades from different hosts and regions has not been fully examined.

As shown by examining the H1N1 lineage evolution within the genetic distance and time dimension (Figure 1), H1 had been circulating in humans for several years before 2009 (Figures 1 and 4a), but when H2 appeared with the outbreak of pdm09, H1 quickly disappeared (Figures 1 and 4a,b). This supports the previous finding that pdm09 replaced the previous seasonal H1N1 (Krammer et al., 2018), likely due to the strong competition between H1 and H2. As compared to other swine lineages (S2, S3), the genetic distance between S1 and avian lineage A1 is closer, but the stop codon is different between them; thus, we speculated that the S1 lineage may have come from the A1 lineage a long time ago. Since the sequences of H1 and S2 have the same codon deletion at the same position, and this deletion does not exist in other lineages, S2 could derive from H1, suggesting another interspecific transmission event (Figure 1, Table 2). H1 disappeared in 2009 due to the emergence and replacement of pdm09 (Krammer et al., 2018), but the S2 lineage was not replaced, likely because lineages in swine suffered less competition and swine became the reservoir of this lineage. Birds, especially aquatic birds, are considered to be the natural reservoir of influenza viruses (Yoon et al., 2014). However, our analysis shows that H1N1 has only one lineage (A1) in birds, and A1 is genetically distant from other lineages and has no genetic crossover with the other lineages (Figures 1 and 2), probably due to the large host barrier between avian and mammals.

Our conclusion regarding the global dynamics has several limitations. First, competition was defined based on co‐occurrence probability rather than direct evidence of causal mechanisms. The mechanism of competition of influenza viruses for antigenic resources needs to be examined and tested. Besides, human intervention such as vaccination, travel restriction, or isolation may attribute to the strength of competition or replacement of lineages. Second, sampling bias may affect our results. Although resampling could help to overcome the problem, many regions are lacking samples which would cause biased estimation of cross‐region transmission or even evolutionary trees. Third, there are more than one hundred subtypes of influenza viruses, and their transmission patterns may be different from H1N1. It is necessary to test the hypotheses using various subtypes.

In summary, we found lineages infecting the same host or hosts with a higher cross‐regional transmission intensity suffered a higher competition and extinction pressure, which highlights the roles of host and geographic barriers in shaping the competition, coexistence, and extinction patterns of H1N1 lineages. Our results suggest that it is necessary to reduce close contact among different hosts to reduce interspecific transmissions and to reduce cross‐border transport of live livestock and poultry to reduce cross‐region transmissions in the world.

## CONFLICT OF INTEREST

The authors declare no conflict of interest.

## AUTHOR CONTRIBUTIONS

**Chaoyuan Cheng:** Conceptualization (equal); Data curation (equal); Formal analysis (equal); Investigation (equal); Methodology (equal); Software (lead); Visualization (lead); Writing – original draft (equal); Writing – review & editing (equal). **Marcel Holyoak:** Writing – review & editing (equal). **Lei Xu:** Writing – review & editing (equal). **Jing Li:** Writing – review & editing (equal). **Wenjun Liu:** Writing – review & editing (equal). **Nils Chr. Stenseth:** Writing – review & editing (equal). **Zhibin Zhang:** Conceptualization (equal); Formal analysis (equal); Funding acquisition (lead); Investigation (equal); Methodology (equal); Project administration (lead); Supervision (lead); Writing – original draft (equal); Writing – review & editing (equal).

## Supporting information

Figure S1

Supplementary Material

## Data Availability

The data that support the findings of this study are openly available in Dryad at https://doi.org/10.5061/dryad.r2280gbcw. The python scripts used to calculate the coexistence coefficient between different lineages and hosts used in this study are openly available in Zenodo at https://doi.org/10.5281/zenodo.4699076.
