# Supplementary figures and images for: Host and geographic barriers shape the competition, coexistence, and extinction patterns of influenza A (H1N1) viruses
Source: Ecol Evol. 2022 Mar 21;12(3):e8732. doi: 10.1002/ece3.8732 (PMC8938227; doi:10.1002/ece3.8732)

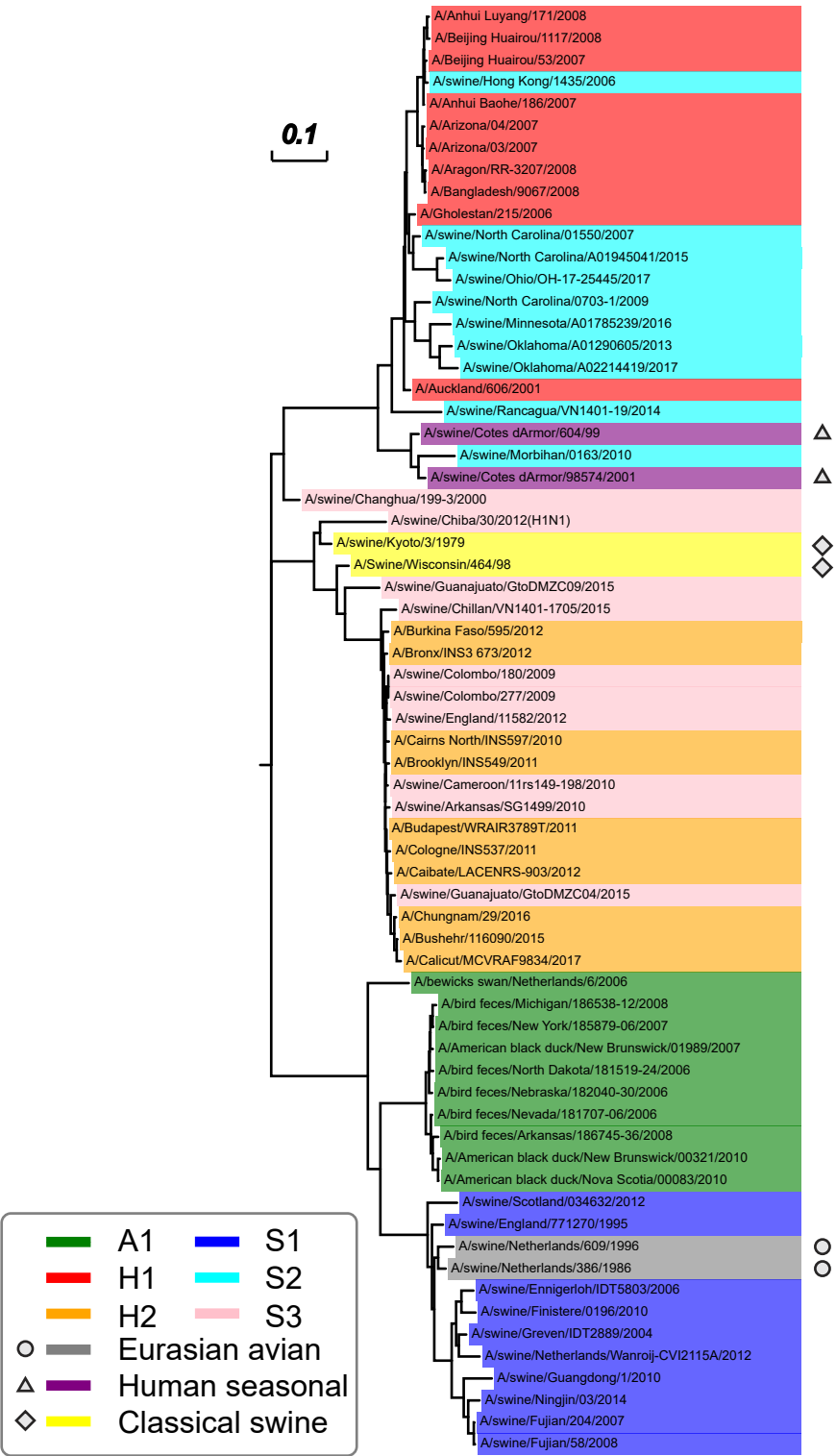

Supplement: Supplementary file 1 — Figure S1 [file ECE3-12-e8732-s002.pdf]
