# Supplementary material for: Host and geographic barriers shape the competition, coexistence, and extinction patterns of influenza A (H1N1) viruses
Source: Ecol Evol. 2022 Mar 21;12(3):e8732. doi: 10.1002/ece3.8732 (PMC8938227; doi:10.1002/ece3.8732)
Supplement: Supplementary file 2 — Supplementary Material [file ECE3-12-e8732-s001.docx]

Supplementary Materials

Figure S1


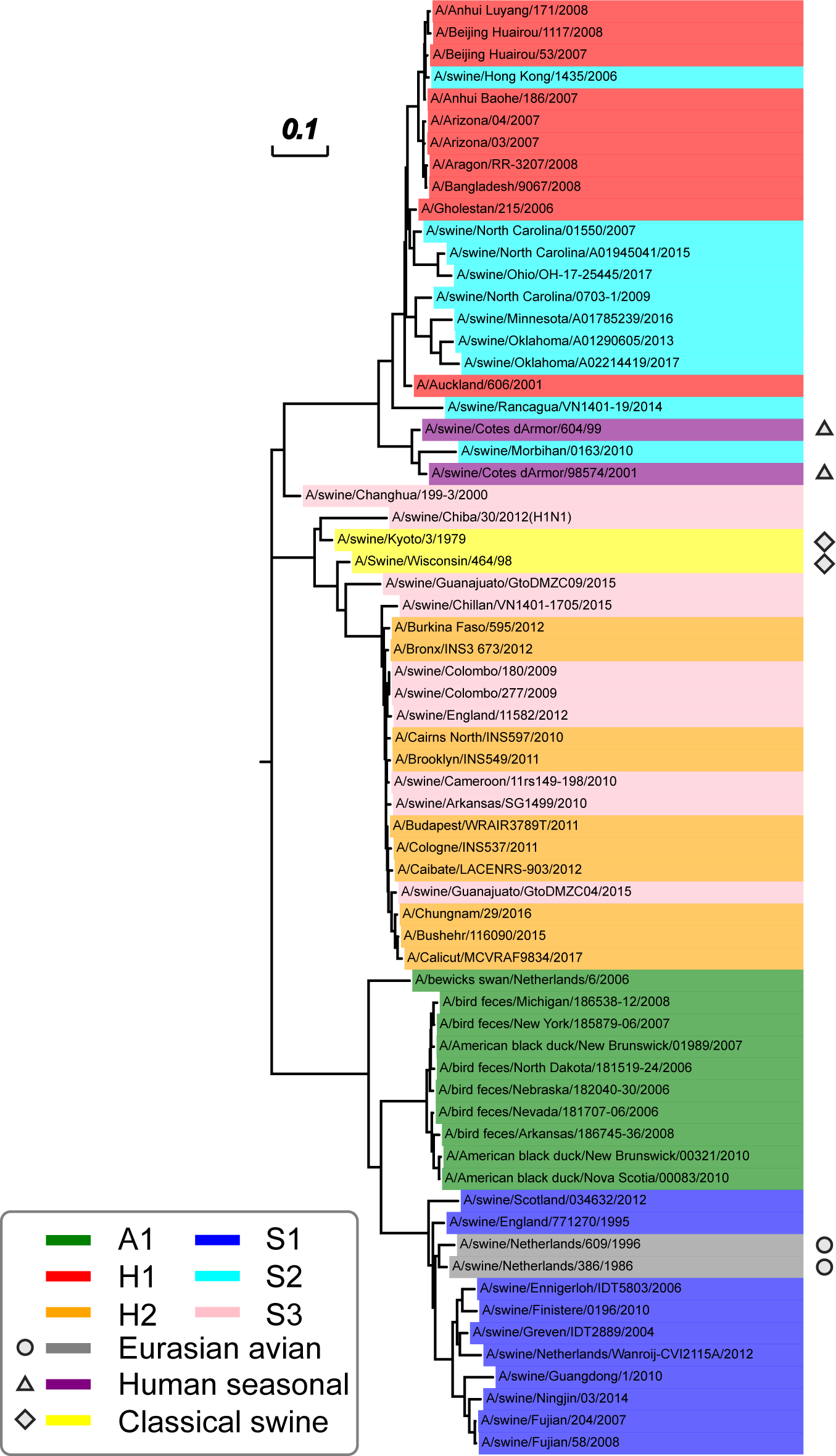


**Fig. S1. The genetic relationship between the lineages we identified (H1, H2, S1, S2, S3, A1) and the generally considered swine influenza A lineages (Eurasian avian, Human seasonal, Classical swine).** Different colors indicate different influenza A (H1N1) lineages.

**Table S1. Statistic values calculated by network analysis using resampled data.**

| Region | Degree | Degree centrality | Closeness centrality | Betweenness centrality | Lineage |
| --- | --- | --- | --- | --- | --- |
| AF | 0 | 0 | 0 | 0 | A1 |
| EU | 1 | 0.125 | 0 | 0 | A1 |
| NAm | 3 | 0.375 | 0.125 | 0.0357 | A1 |
| NEA | 1 | 0.125 | 0.1667 | 0 | A1 |
| OC | 0 | 0 | 0 | 0 | A1 |
| SAm | 0 | 0 | 0 | 0 | A1 |
| SA | 0 | 0 | 0 | 0 | A1 |
| SEA | 0 | 0 | 0 | 0 | A1 |
| WA | 1 | 0.125 | 0.1667 | 0 | A1 |
| AF | 3 | 0.375 | 0.4091 | 0.0446 | H1 |
| EU | 10 | 1.25 | 0.3750 | 0.3095 | H1 |
| NAm | 1 | 0.125 | 0.3224 | 0 | H1 |
| NEA | 8 | 1 | 0.5625 | 0.2202 | H1 |
| OC | 5 | 0.625 | 0.5000 | 0.1250 | H1 |
| SAm | 3 | 0.375 | 0.4091 | 0.0060 | H1 |
| SA | 2 | 0.25 | 0.4712 | 0 | H1 |
| SEA | 2 | 0.25 | 0.3 | 0.0000 | H1 |
| WA | 6 | 0.75 | 0.5 | 0.2054 | H1 |
| AF | 9 | 1.125 | 0.4083 | 0.4613 | H2 |
| EU | 3 | 0.375 | 0.4375 | 0.1458 | H2 |
| NAm | 8 | 1 | 0.2917 | 0.0298 | H2 |
| NEA | 6 | 0.75 | 0.6125 | 0.3571 | H2 |
| OC | 3 | 0.375 | 0.3403 | 0.0000 | H2 |
| SAm | 1 | 0.125 | 0.3478 | 0 | H2 |
| SA | 5 | 0.625 | 0.4375 | 0.1369 | H2 |
| SEA | 5 | 0.625 | 0.5104 | 0.2054 | H2 |
| WA | 4 | 0.5 | 0.4712 | 0.0208 | H2 |
| AF | 0 | 0 | 0 | 0 | S1 |
| EU | 2 | 0.25 | 0.125 | 0 | S1 |
| NAm | 0 | 0 | 0 | 0 | S1 |
| NEA | 2 | 0.25 | 0.125 | 0 | S1 |
| OC | 0 | 0 | 0 | 0 | S1 |
| SAm | 0 | 0 | 0 | 0 | S1 |
| SA | 0 | 0 | 0 | 0 | S1 |
| SEA | 0 | 0 | 0 | 0 | S1 |
| WA | 0 | 0 | 0 | 0 | S1 |
| AF | 0 | 0 | 0 | 0 | S2 |
| EU | 2 | 0.25 | 0.1875 | 0.0089 | S2 |
| NAm | 3 | 0.375 | 0.2813 | 0.0714 | S2 |
| NEA | 3 | 0.375 | 0.225 | 0.0714 | S2 |
| OC | 0 | 0 | 0 | 0 | S2 |
| SAm | 2 | 0.25 | 0.1875 | 0.0089 | S2 |
| SA | 0 | 0 | 0 | 0 | S2 |
| SEA | 0 | 0 | 0 | 0 | S2 |
| WA | 0 | 0 | 0 | 0 | S2 |
| AF | 2 | 0.25 | 0.2500 | 0 | S3 |
| EU | 1 | 0.125 | 0.1667 | 0.0000 | S3 |
| NAm | 3 | 0.375 | 0.1250 | 0 | S3 |
| NEA | 8 | 1 | 0.125 | 0.0893 | S3 |
| OC | 1 | 0.125 | 0.1667 | 0 | S3 |
| SAm | 1 | 0.125 | 0.1667 | 0.0000 | S3 |
| SA | 1 | 0.125 | 0.1667 | 0 | S3 |
| SEA | 1 | 0.125 | 0.1667 | 0 | S3 |
| WA | 0 | 0 | 0 | 0 | S3 |
